# Exercise Factors Released by the Liver, Muscle, and Bones Have Promising Therapeutic Potential for Stroke

**DOI:** 10.3389/fneur.2021.600365

**Published:** 2021-05-24

**Authors:** Joseph S. Stephan, Sama F. Sleiman

**Affiliations:** ^1^School of Medicine, Lebanese American University, Byblos, Lebanon; ^2^Biology Program, Lebanese American University, Byblos, Lebanon

**Keywords:** exercise factors, beta-hydroxybutyrate, irisin, lactate, BDNF, stroke, osteocalcin

## Abstract

Stroke is one of the leading causes of death and disability in the world. Stroke not only affects the patients, but also their families who serve as the primary caregivers. Discovering novel therapeutic targets for stroke is crucial both from a quality of life perspective as well as from a health economic perspective. Exercise is known to promote neuroprotection in the context of stroke. Indeed, exercise induces the release of blood-borne factors that promote positive effects on the brain. Identifying the factors that mediate the positive effects of exercise after ischemic stroke is crucial for the quest for novel therapies. This approach will yield endogenous molecules that normally cross the blood brain barrier (BBB) and that can mimic the effects of exercise. In this minireview, we will discuss the roles of exercise factors released by the liver such as beta-hydroxybutyrate (DBHB), by the muscle such as lactate and irisin and by the bones such as osteocalcin. We will also address their therapeutic potential in the context of ischemic stroke.

## Introduction

Stroke is the fifth major cause of death and a leading cause of disability in the United States. This is due to the lack of neuroprotective agents that are able to decrease the associated neuronal damage and loss (1, 2). Neurotrophins such as brain-derived neurotrophic factor (BDNF) mediate protection and recovery following stroke. BDNF promotes regeneration and restores damaged neural tissue. The use of exogenous BDNF after stroke is hindered by its rapid degradation and its inability to cross the blood-brain barrier (BBB). Hence, therapeutics that can modulate endogenous BDNF signaling in the brain may be useful in the context of stroke (3).

**Figure 1:**
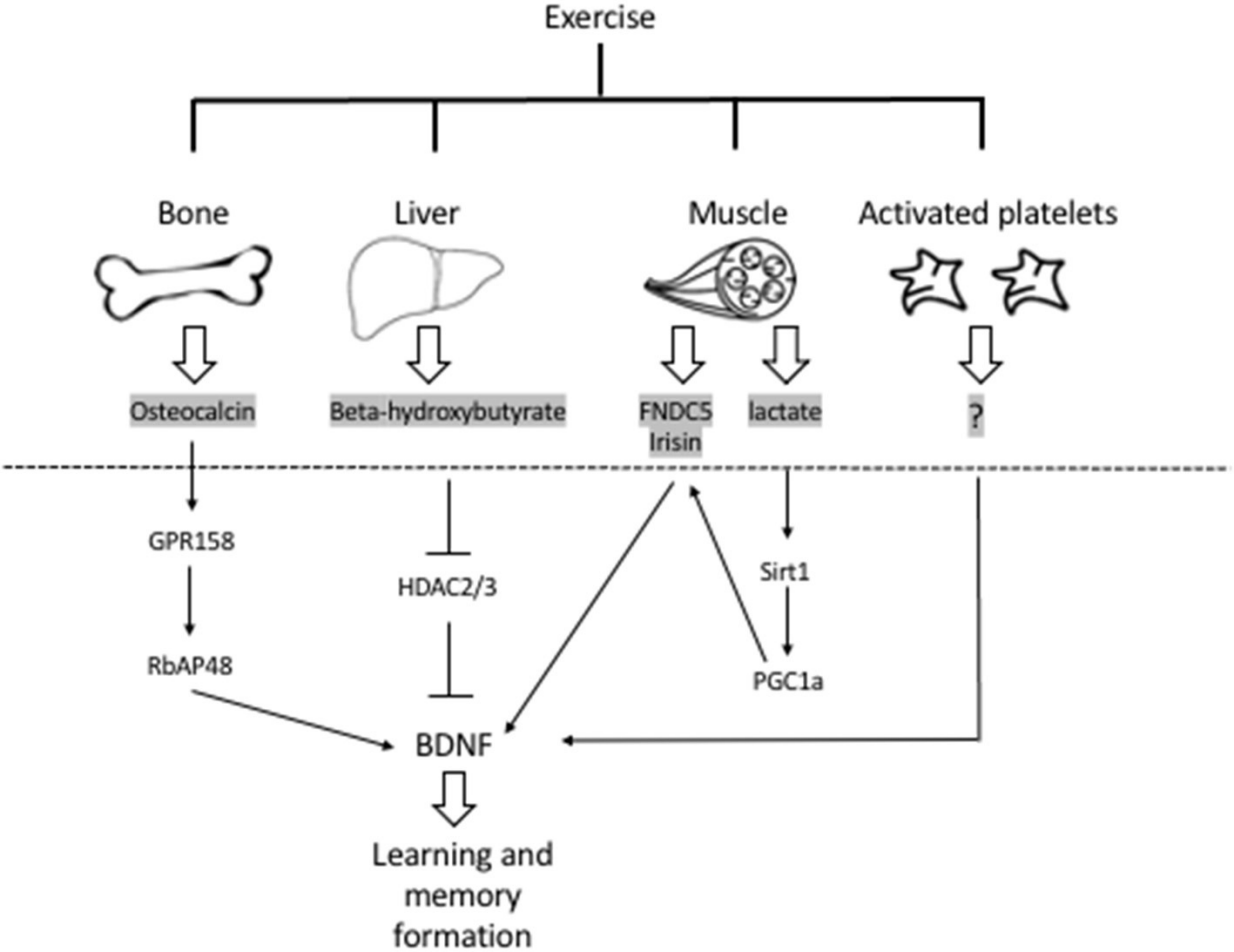
Exercise induces the release of metabolites and proteins that promote learning and memory formation through activation of *Bdnf* expression and signaling in the hippocampus. Bones release osteocalcin that promotes *Bdnf* expression through an epigenetic mechanism involving RbAP48 (5, 6). The liver releases the ketone body betahydroxy-butyrate that induces hippocampal *Bdnf* expression by inhibiting class I HDACs, namely HDAC2 and HDAC3 (4, 7). The muscle releases FNDC5/irisin that activate hippocampal *Bdnf* expression through an unknown mechanism (8). In addition, the muscle release lactate that activates the hippocampal SIRT1/PGC1-alpha/FNDC5 pathway and in turn *Bdnf* expression (9). Finally activated platelets have been shown to store and secrete BDNF (10, 11). Whether these factors mediate the positive effects of exercise in stroke is not clear and needs to be addressed. In addition, the role of BDNF signaling in exercise-mediated neuroprotection also needs to be assessed. BDNF, brain-derived neurotrophic factor; FDNC5, fibronectin type III domain-containing protein 5; HDACs, histone deacetylases; SIRT1, sirtuin 1; PGC1-alpha, Peroxisome proliferator-activated receptor gamma coactivator 1-alpha.

Physical exercise increases *Bdnf* expression in the hippocampus to promote learning, and memory formation (4). Exercise mediates these positive effects by inducing the release of metabolites and proteins from the liver, muscle, bones and platelets (Figure 1). These factors have been shown to be protective in the context of traumatic brain injury (12) and depression (13). In addition, exercise can prevent and alleviate many of the detrimental effects of stroke. Understanding which factors mediate the protective effects of exercise in ischemic stroke and deciphering the involvement of BDNF signaling will allow us to fully harness exercise's therapeutic potential.

In this minireview, we will focus on how newly identified blood-borne exercise factors that induce hippocampal BDNF signaling to promote learning and memory formation are protective after ischemic stroke. We will also address the current research gaps that link these factors to the positive effects of exercise. Indeed, this minireview will highlight the urgent need for systemic experimentation to identify which factors are responsible for exercise's prophylactic and therapeutic effects in the context of stroke and the role of BDNF signaling in these effects.

### Exercise and Ischemic Stroke

It is important to distinguish between the preventative roles of exercise and its therapeutic role. Exercise pre-conditioning enhances neuroprotection and decreases brain edema (14–23). Treadmill exercise prior to middle cerebral artery occlusion (MCAO) in rodent models improves motor function, decreases infarct volumes, reduces neuronal apoptosis and oxidative stress, enhances angiogenesis and induces *Bdnf* expression (24–27). The level of neuroprotection that is achieved varies with the duration and intensity of exercise. For example, even though short bouts of exercise before stroke induce the expression of angiogenesis markers, they may not be enough to rescue neurological deficits post-ischemic stroke (26). Alternatively, high intensity interval training (HIIE) alleviates the symptoms of stroke more efficiently than moderate continuous training (28). Moderate exercise may protect the brain against MCAO by enhancing the release of miR-126 enriched endothelial progenitor cell-derived exosomes (29).

Exercise is also a safe and cost-effective therapeutic strategy post-stroke, in which the optimal time and intensity of exercise is critical (30). Indeed, animal studies have revealed that both high intensity exercise or exercise initiated only 24 h after stroke promote inflammation and cell death (30). In contrast, exercise initiated on later time periods (as early as 48 h post-stroke) as well as low intensity and moderate intensity exercise improve infarct volume and neurological severity scores 14 days post-stroke (30). Interestingly, prolonged treadmill exercise promotes neurogenesis and improves motor function and short-term memory by increasing the expression of hippocampal BDNF in photothrombotic stroke mice (31). In addition, treadmill exercise enhances neurogenesis and myelin repair by activating the Wnt and BDNF pathways after focal cerebral ischemia/reperfusion (32).

In most of the paradigms in which exercise was studied as a prophylactic (14–23) or as a therapy for ischemic stroke (33–37), the neuroprotective roles of exercise were associated with restoration of BDNF levels (38, 39). In humans, decreases in BDNF levels are correlated with an increased risk of stroke, worse functional outcomes and higher mortality (40). Indeed, BDNF levels are decreased in acute ischemic-stroke patients. Interestingly, patients that carry the BDNF Val^66^Met allele, known to decrease BNDF levels by 30%, have worse outcomes and prognosis after stroke (40).

### Exercise Factors and the Brain

Exercise enhances neurogenesis, mediates synaptic plasticity and promotes learning and memory formation. These effects are thought to be mediated in part by activation of hippocampal BDNF signaling [reviewed in (4)]. Recent work has revealed that injection of blood from young exercising mice is able to rescue learning and memory defects in old mice by inducing hippocampal BDNF levels (41). Several blood-borne exercise factors comprised of proteins and metabolites released by the liver, muscle and bones have been identified through their ability to induce BDNF signaling, and have been implicated in mediating the positive effects of exercise on the brain. Since decreases in BDNF levels are correlated with negative outcomes after stroke (40), studying the exercise factors that induce BDNF signaling and assessing their neuroprotective abilities may allow us to identify novel endogenous therapeutic agents for stroke. For this reason, we will discuss what is known about blood-borne factors released by the liver, muscle and bones. Interestingly, even though some of these factors have neuroprotective effects in animal models of stroke, very little has been done to directly demonstrate that they are responsible for the preventative or therapeutic effects of exercise. These studies remain necessary to further our understanding of the molecular mechanisms underlying the effects of exercise.

### Exercise Factors Released by the Liver and Their Role in Ischemic Stroke

Exercise induces the release of multiple factors from the liver into the blood that can transfer its benefits to the brain. These exercise factors include metabolites, such as the ketone body beta-hydroxybutyrate (DBHB) (7) and proteins, such as glycosylphosphatidylinositol (GPI)–specific phospholipase D1(Gpld1)(41).

#### Beta-Hydroxybutyrate (DBHB)

During exercise, the liver releases DBHB into the blood. DBHB crosses the BBB and accumulates in the hippocampus, where it induces *Bdnf* expression by acting as a class I histone deacetylase inhibitor (7). Multiple studies have demonstrated the beneficial effects of ketone bodies and ketogenic diets for brain health. Ketogenic diets rescue neurogenesis defects and prevent memory abnormalities in Kabuki syndrome by inducing transcriptional changes through histone deacetylase (HDAC) inhibition (42). They also extend longevity, improve memory and enhance brain health in aging mice (43, 44). DBHB improves multiple cellular pathologies in Parkinson's disease (PD) [reviewed in (45)] and improves learning and memory formation in a mouse model of Alzheimer's disease (AD) (46). In addition, DBHB has antidepressant effects: it decreases depressive behaviors in mice by increasing histone3-lysine9-β-hydroxybutyrylation and promoting BDNF expression (47). Interestingly, DBHB and ketogenic diets also have promising neuroprotective potential against stroke.

Both DBHB and ketogenic diets promote neuroprotection after stroke. They decrease infarct volume after permanent and transient MCAO (48, 49). DBHB improves cerebral energy metabolism during ischemia and inhibits lipid peroxidation after reperfusion (49). A ketogenic diet improves ischemic tolerance to MCAO and inhibits the nucleotide-binding domain (NOD)-like receptor protein 3 (NLRP3) inflammasome in the brain (50). DBHB also inhibits dynamin-related protein 1 (Drp1)-mediated mitochondrial fission and suppresses endoplasmic reticulum stress-activated NLRP3 inflammasome in oxygen-glucose deprived (OGD) neuroblastoma cells (50). This later pathway is involved in detecting cellular damage and mediating inflammation during ischemic stroke. Combined treatment of DBHB with another ketone body, acetoacetate decreases infarct volume, improves neurologic function, and increases the NAD^+^/NADH ratio, Sirtuin 3 (Sirt3), Forkhead Box O3a, and Superoxide Dismutase 2 expression in the penumbra (51). Interestingly, knockdown of Sirt3 in primary neurons attenuates the ability of ketone bodies to promote cell survival in a rotenone-dependent model of neuronal death, suggesting that SIRT3 may mediate the pro-survival effects of ketone bodies (51). More work is needed to fully decipher this pathway and to assess its contribution to neuroprotection *in vivo*.

**Figure 2:**
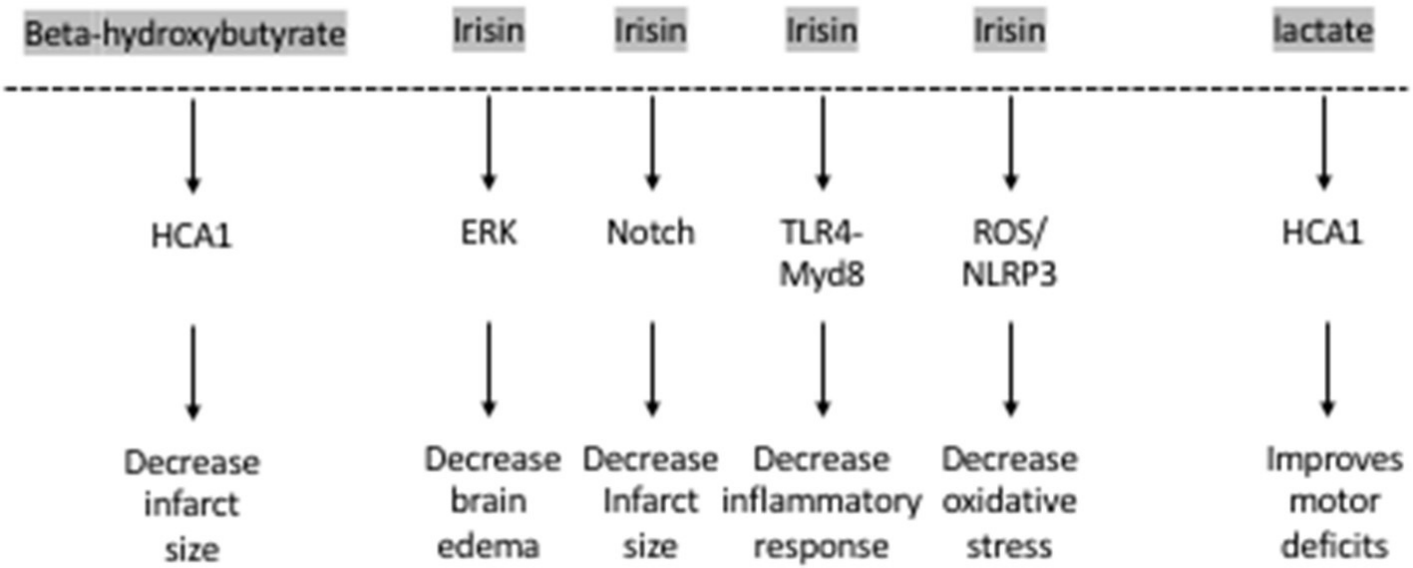
The exercise factors, beta-hydroxybutyrate, irisin and lactate protect against ischemic stroke.

The hydroxy-carboxylic acid receptor 2 (HCA2) mediates the neuroprotective effect of ketogenic diets and DBHB in cerebral ischemia (52) (Figure 2). Indeed, while both ketogenic diets and DBHB significantly decrease infarct size after MCAO, this protective effect is lost in the HCA2 knockout mice despite higher plasma levels of ketone bodies (52). Interestingly, the HCA2 protective effect is mediated by infiltrating macrophages and monocytes, as activation of HCA2 in these cells is neuroprotective (52). This work suggests that the DBHB released from the liver mediates neuroprotection by modulating neuroinflammation. As a result, it is important to understand whether DBHB also activates its receptors in neuronal cells after ischemic stroke to mediate its neuroprotective role. Tissue-specific knockouts of this receptor will help determine whether its roles are restricted to immune cells or whether it plays important signaling effects in neurons. More studies are also needed to determine whether DBHB is responsible for mediating exercise's neuroprotective effects in cerebral ischemia, and to identify the molecular mechanism underlying these neuroprotective effects. Since we already know that exercise increases DBHB levels in the hippocampus where it increases *Bdnf* expression through HDAC inhibition (7) and that DBHB induces resistance to oxidative stress *via* HDAC inhibition (53), it is important to assess whether epigenetic mechanisms are involved in DBHB's neuroprotective effects considering the efficacy of HDAC inhibition as a therapy in mouse models of stroke (54, 55).

#### Glycosylphosphatidylinositol–Specific Phospholipase D1(Gpld1)

In addition to ketone bodies, exercise induces the liver to release multiple proteins into the blood. A recent study has revealed that exercise increases the levels of a GPI- degrading enzyme, Gpld1, in the blood of mice and of healthy elderly humans (41). Interestingly, unlike other exercise factors discussed in this minireview, liver-derived Gpld1 does not readily enter the brain (41). However, overexpression of Gpld1 in the liver of aged mice increases its levels in the plasma, significantly induces BDNF protein expression in the hippocampus and rescues impaired neurogenesis as well as age-dependent cognitive impairments observed in the radial arm water maze, Y maze and novel object recognition paradigms (41). Coagulation and complement signaling are the major cascades that are altered in response to Gpld1 overexpression and exercise. These cascades were suggested to be involved in mediating Gpld1 and exercise's effects on neurogenesis and cognition (41). Interestingly, previous work has shown that exercise activates platelets and that activated platelets promote neurogenesis by releasing factors such as platelet factor 4 (10). Indeed, platelet depletion abolishes exercise-induced neurogenesis in the hippocampus (10). It would be important to determine whether liver-derived Gpld1 is involved in the exercise-mediated platelet responses. Interestingly, in the context of stroke, Gpld1 was identified as a critical regulator of platelet activity (56). Both pharmacological inhibition and genetic ablation of Gpld1 protected from pathological thrombus formation and ischemic stroke (56, 57). As a result, Glpd1 mediates both positive and negative effects on the brain depending on context. Resolving the contradictory roles played by Gpld1is necessary and can be achieved by studying the role of exercise-induced Gpld1in stroke mouse models. This will help in determining whether this protein is involved in mediating the neuroprotective effects of exercise in stroke or whether its neuroprotective roles are restricted to healthy subjects.

### Exercise Factors Released by the Muscle and Their Role in Stroke

In addition to the liver, exercise induces the muscle to release multiple factors that have been shown to play important roles in regulating brain health by regulating BDNF levels. These include proteins such as Cathepsin B, FNDC5, and its cleavage product irisin as well as metabolites such as lactate.

#### Cathepsin B

Cathepsin B (CTSB), a lysosomal cysteine protease, is released by the muscle during exercise. It mediates the positive effects of exercise on cognition by increasing adult hippocampal neurogenesis and promoting spatial memory formation (58, 59). Indeed, CTSB increases BDNF and doublecortin (DCX) levels in adult hippocampal progenitor cells (58). Exercise also increases CTSB levels in the plasma of humans, where its levels are correlated with hippocampus-dependent memory formation (58). CTSB also controls neurite outgrowth by modulating lysosomal trafficking in neurons (60). In humans, the effects of exercise on plasma CTSB levels are variable with some studies showing that long-term treadmill running increase plasma CTSB levels (58), while others showing no changes in plasma CTSB levels after 6 weeks of HIIE, or a single bout of HIIE in sedentary males (61, 62). Interestingly, studies that focus on the role of muscle-released CTSB in cerebral ischemia are sparse. In contrast, a negative role for brain-derived CTSB in cerebral ischemia has been established. For example, both genetic ablation and pharmacological inhibition of CTSB in mice protect hippocampal neurons from hypoxic/ischemic injury (63) and significantly decrease reactive oxygen species (ROS) production and neuroinflammation (64). CTSB mediates the neurotoxic polarization of microglia/macrophages, worsening hypoxia/ischemia-induced neuronal injury (63). Moreover, CTSB mediates secondary degeneration in the ipsilateral thalamus and substania nigra after focal cortical infarction (65, 66). Based on what is known, CTSB is unlikely to mediate the neuroprotective effects of exercise in stroke patients. However, more work is needed to identify which exercise paradigms consistently induce release of CTSB from the muscle and to determine whether the role of the muscle-released CTSB protein is distinct from the neuronal protein.

#### Fibronectin Type III Domain-Containing Protein 5 and Its Cleavage Product Irisin

Other myokines such as the Fibronectin type III domain-containing protein 5 (FNDC5) and its secreted cleavage product, irisin, also mediate the positive effects of exercise on the brain by inducing hippocampal *Bdnf* expression (8, 67). Lactate, a metabolite released by the muscle during exercise, crosses the blood-brain barrier and activates the hippocampal PGC1a/FNDC5 pathway (9). Lactate increases the levels and activity of the lysine deacetylase Sirtuin 1(SIRT1). SIRT 1 activates the transcriptional activation complex PGC-1alpha/ERRa, which increases hippocampal *Fndc5* expression. FNDC5, in turn, activates *Bdnf* expression, promoting learning and memory formation (9). Moreover, peripheral delivery of FNDC5 increases blood irisin levels, and also induces hippocampal *Bdnf* expression (8). In addition to mediating exercise's positive effects on cognition, these proteins have been shown to rescue cognitive deficits associated with neurodegenerative diseases such as Alzheimer's disease (68, 69) by inducing BDNF signaling (70) as well as behavioral deficits observed in mouse models of depression (71, 72). Irisin's antidepressant effect also involves modulation of *Bdnf* expression (73).

Interestingly, irisin plays important neuroprotective roles. The levels of irisin in the blood decrease after ischemic stroke in mouse models (74) and in humans (75). Decreased irisin levels were associated with poor prognosis in patients who have suffered from an ischemic stroke (75) (Figure 2). Irisin decreases brain edema through the ERK pathway, decreases infarct size through the Notch pathway, decreases oxidative stress through the TLR4/Myd8 pathway and decreases the inflammatory response through the ROS/NLP3 pathway (76). Indeed, irisin is neuroprotective both *in vitro* and *in vivo*. Irisin administration protects against OGD-induced neuronal death *in vitro* (74) in part by inhibiting the ROS-NLRP3 signaling pathway (77). Moreover, irisin protects against damage induced by a cerebral ischemia/reperfusion (I/R) model by modulating the Notch signaling pathway (78). Irisin treatment decreases the infarct size, brain edema and neurological deficits in mice subjected to MCAO (74). This irisin-mediated rescue of brain damage is associated with decreased apoptosis and increased cortical levels of BDNF (79). It is thought that the Akt and ERK1/2 pathways, known to be downstream effectors of BDNF signaling, mediate irisin's neuroprotective effects (74). Interestingly, 3 weeks of high intensity training resulted in increased BDNF in the brain and plasma following MCAO and this increase was dependent on the PGC-1a pathway (80). Based on these observations, both FNDC5 and irisin are likely involved in mediating the neuroprotective effects of exercise against ischemic stroke. More work is needed to establish this direct link by assessing the neuroprotective effects of exercise in *Fndc5* knockout mice.

#### Lactate

Another exercise factor that is released by the muscle is lactate. It is well-established that lactate is used as an energy substrate by the brain (81) and that neuronal uptake of astrocytic lactate is required for long-term memory formation (82). Indeed, RNA sequencing data reveals that lactate increases the expression of both neuroprotective and synaptic plasticity genes such as *Bdnf* , *Arc, c-Fos*, and *Zif268* by inducing NMDA receptor activity and its downstream signaling pathway Erk1/2 in primary neuronal cultures and in cortical tissues (83, 84). We only recently identified lactate as a muscle-released exercise factor that enhances spatial memory by activating the PGC1a/FNDC5/BDNF signaling pathway in the hippocampus (9). Like other exercise factors, lactate promotes brain health and rescues from a variety of central nervous system (CNS) disorders. It enhances neurogenesis by activating the NF-kB signaling pathway following intracerebral hemorrhage (85) and rescues cognitive defects in mice subjected to fluid percussion injury [reviewed in (12)]. It also acts as an antidepressant (13, 86). Interestingly, lactate also has extensive neuroprotective roles.

Lactate mediates neuroprotection against glutamate-mediated excitotoxicity in mouse cortical neurons by engaging a network of cellular pathways involving ATP production, and activation of KATP channels (87). It also promotes resistance to H_2_O_2_-induced death in neuroblastoma cells by activating the Unfolded Protein Response (UPR) and nuclear factor erythroid 2-related factor 2 (NRF2) (88). In addition, lactate protects against OGD-induced neuronal death in rat organotypic hippocampal slices (89, 90). This pro-survival effect involves increasing the expression of the potassium channel TREK1 by activating the PKA pathway in astrocytes during ischemia (91).

Lactate also enhances neuroplasticity post-stroke. Both intracerebroventricular and systemic injections of lactate directly after reperfusion improve neurologic outcomes 48 h after cerebral ischemia (89, 90). These beneficial effects of lactate appear to be long-lasting. Improved neurological scores in the rotarod test and the beam walking test can still be observed 2 weeks after ischemia in mice subjected to MCAO and receiving intraventricular injections of lactate after reperfusion (89). There is evidence that the HCA1 receptor may mediate lactate's protective effects in neurons of the ischemic cortex after MCAO (92) (Figure 2).

Taken together, the data suggest that lactate plays important neuroprotective roles and enhances positive functional outcomes after stroke. Direct evidence that lactate is mediating exercise's protective roles after ischemic stroke remain elusive. Genetic or pharmacological inhibition of the monocarboxylate transporters (MCT2), particularly the MCT2, may aid in understanding whether both lactate and DBHB mediate exercise's neuroprotective effects after cerebral ischemia.

### Exercise Factors Released by the Bones and Their Role in Stroke

#### Osteocalcin

While traditionally regarded as a structural organ, bone has attracted attention in recent years for its endocrine functions. One protein released by osteoblasts in response to endurance exercise in mice and humans is osteocalcin (OCN) (93). A single bout of HIIE in healthy male individuals increased corticospinal excitability, BDNF and uncarboxylated OCN (uncOCN). Indeed, greater increases in BDNF were linked to increases in unOCN and irisin only in the exercising individuals, suggesting that these factors may contribute to exercise-induced BDNF increases (61). Interestingly, OCN delivery was previously shown to be sufficient to improve memory and decrease anxiety-like behaviors in aging mice (94). These positive effects of OCN were mediated by directly increasing hippocampal BDNF levels through activation of the Gpr158, an orphan G protein-coupled receptor (5).

Recent studies have shown that OCN enhances neuroplasticity by improving outcomes after ischemic stroke (95). Stroke patients who had better outcomes had higher serum osteocalcin levels than those whose National Institutes of Health Stroke Scale (NIHSS) scores did not improve. At the molecular level, metabolic reprogramming and decreased pyroptosis were responsible for the neuroprotective effect of OCN in an OGD model (95). Even though current work has not shown that OCN directly mediates the beneficial effects of exercise in the brain, the available evidence suggests that it may be a candidate exercise factor that is worth assessing in animal models of stroke.

## Discussion

Exercise has profound positive effects on the brain including induction of synaptic plasticity, neurogenesis and enhancement of learning and memory formation. The positive effects of exercise are thought to be mediated by multiple exercise factors that induce BDNF signaling. Exercise is also effective in ameliorating the detrimental symptoms of ischemic strokes in animal models and in humans. Even though some of the molecular pathways underlying the neuroprotective effects of exercise are known, it is clear that not all the currently known exercise factors are involved in mediating these effects (Figure 2). It is important to conduct a systemic analysis to identify which exercise-induced blood-borne factors, alone or in combination, mediate exercise's neuroprotective effects in cerebral ischemia. Indeed, systematic studies assessing how the frequency, intensity, and duration of the exercise impact its ability to enhance the production of these blood-borne factors are needed. Moreover, it is important to understand how the levels of these factors are modulated when individuals exercise prior or after stroke. These along with experiments designed to assess which exercise factors are responsible for mediating exercise's prophylactic and therapeutic effects in the context of stroke will allow us to develop targeted therapeutic approaches. Metabolites such as DBHB, lactate and proteins such as irisin, initially identified as exercise factors that induce *Bdnf* expression (7–9) and promote learning and memory formation, are the leading candidates of the currently known exercise factors (Figure 1).

## Author Contributions

JS and SS wrote and edited the manuscript. All authors contributed to the article and approved the submitted version.

## Conflict of Interest

The authors declare that the research was conducted in the absence of any commercial or financial relationships that could be construed as a potential conflict of interest.
